# From thymus to periphery: Molecular basis of effector γδ‐T cell differentiation

**DOI:** 10.1111/imr.12918

**Published:** 2020-11-15

**Authors:** Gina J. Fiala, Anita Q. Gomes, Bruno Silva‐Santos

**Affiliations:** ^1^ Instituto de Medicina Molecular João Lobo Antunes Faculdade de Medicina Universidade de Lisboa Lisbon Portugal; ^2^ H&TRC Health & Technology Research Center ESTeSL—Escola Superior de Tecnologia da Saúde Instituto Politécnico de Lisboa Lisbon Portugal

**Keywords:** effector T cell differentiation, Gamma‐delta T cells, IL‐17, Thymic T cell development

## Abstract

The contributions of γδ T cells to immune (patho)physiology in many pre‐clinical mouse models have been associated with their rapid and abundant provision of two critical cytokines, interferon‐γ (IFN‐γ) and interleukin‐17A (IL‐17). These are typically produced by distinct effector γδ T cell subsets that can be segregated on the basis of surface expression levels of receptors such as CD27, CD44 or CD45RB, among others. Unlike conventional T cells that egress the thymus as naïve lymphocytes awaiting further differentiation upon activation, a large fraction of murine γδ T cells commits to either IFN‐γ or IL‐17 expression during thymic development. However, extrathymic signals can both regulate pre‐programmed γδ T cells; and induce peripheral differentiation of naïve γδ T cells into effectors. Here we review the key cellular events of “developmental pre‐programming” in the mouse thymus; and the molecular basis for effector function maintenance vs plasticity in the periphery. We highlight some of our contributions towards elucidating the role of T cell receptor, co‐receptors (like CD27 and CD28) and cytokine signals (such as IL‐1β and IL‐23) in these processes, and the various levels of gene regulation involved, from the chromatin landscape to microRNA‐based post‐transcriptional control of γδ T cell functional plasticity.

## 1 INTRODUCTION: GENERATING SUBSETS OF MURINE γδ T CELLS

The biology of γδ T cells has been extensively studied in mouse models, on which we will focus this review. Such studies have shown that while γδ T cells develop alongside αβ T cells in the thymus, upon thymic egress most γδ T cells atypically localize to non‐lymphoid peripheral tissues, where they can comprise up to 50% of all T cells. In the mouse, the tissue localization of γδ T cells segregates with surface expression of specific T cell receptor (TCR) γ chains. Early work on the genetics of TCR rearrangement during fetal, neonatal and adult thymocyte development revealed an organized and sequential rearrangement of specific *Vγ*‐gene segments in developing γδ T cells during ontogeny. This ordered rearrangement results in timed production of defined γδ T cell populations, which leave the thymus and populate different epithelial‐rich tissues in the adult animal (reviewed in 1). Interestingly, the timely controlled pattern of Vγ chain expression during ontogeny recapitulates the order of *Vγ* organization at the *Cγ1‐TCRγ* locus and proceeds from 3’‐located Vγ5 to 5’‐located Vγ4^2^ (mouse *Vγ* gene nomenclature of 3).

The first T cells produced in the fetal thymus are dendritic epidermal T cells (DETC), a specialized subset of γδ T cells expressing an invariant Vγ5Vδ1 TCR. Subsequently, Vγ6^+^ γδ T cells are generated.^4^, ^5^ Both DETC and Vγ6^+^ γδ T cells develop exclusively in the fetal thymus and have no junctional diversity due to absence of terminal deoxynucleotidyl transferase expression. DETC are found in the adult skin, while Vγ6^+^ γδ T cells localize to diverse tissues such as, eg, uterine epithelia, tongue and meninges.^6^, ^7^, ^8^ Importantly, generation of DETC and Vγ6^+^ γδ T cells is restricted to the confined window of fetal development and cannot be induced in adult animals.^9^, ^10^ Similarly, a subset of NKTγδ T cells expressing a Vγ1Vδ6 TCR is mainly produced in the perinatal phase.^11^ Other Vγ1^+^ and Vγ4^+^ γδ T cells develop from late fetal life onwards, throughout adulthood. These show higher junctional diversity and localize to diverse sites including peripheral lymphoid tissues, where they represent the majority of γδ T cells in the adult mouse. The distinctive developmental phases, orchestration of TCR rearrangements and specialized tissue localization suggest a diversity of physiological roles for γδ T cells encompassing features of both innate and adaptive – or “adaptate” – immune surveillance.^12^

The participation of γδ T cells in immune responses through production of IFN‐γ has long been established, and linked to beneficial roles in many cancer settings (reviewed in 13), as well as in viral, parasitic and intracellular bacterial infections, such as *Listeria monocytogenes*.^14^, ^15^ By contrast, γδ T cells producing IL‐17 (γδ17 T cells) were identified more recently in mice as required for IL‐17‐mediated neutrophil recruitment during the early immune response to *E coli* infection.^16^, ^17^ Since then, γδ17 T cells have been described in diverse tissues as playing either beneficial roles in host defense against *Staphylococcus aureus* and *Candida albicans*, among other infections, or detrimental roles in inflammatory diseases and in cancer, where they can promote angiogenesis, immune suppression and tumor cell growth (reviewed in 13, 18, 19).

**FIGURE 1:**
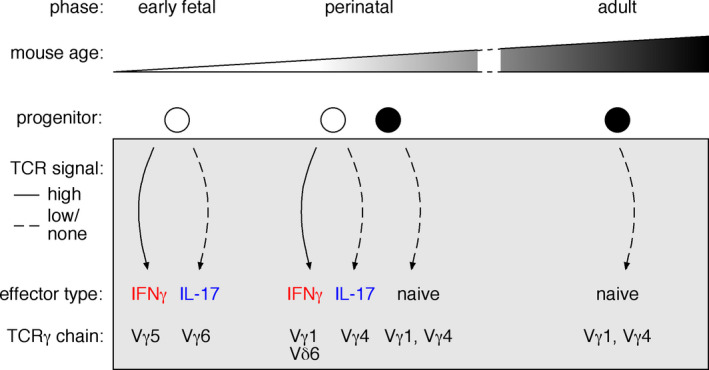
Thymic development of γδ T cells throughout mouse ontogeny. Limited windows in time allow for the generation of specific γδ T cell subsets in the murine thymus. γδ T cell development starts during early fetal and continues throughout life. The combination of distinct progenitors and a changing thymic microenvironment, constrains γδ T cell development during ontogeny. γδ T cells undergo effector cell differentiation inside the thymus influenced by signals received through the TCR. Strong TCR signaling promotes the IFNγ effector program, while IL‐17‐producing γδ T cells develop upon no/weak TCR signals. The dynamic changes of γδ T cell development are further reflected in specific patterns of TCRγ chain rearrangement and expression

The importance of γδ T cell functions mediated by IFN‐γ and IL‐17 has moved the field towards acknowledging two main effector γδ T cell subsets (Figure 1):

1. IFN‐γ‐producing (γδIFN) T cells, including a fetal/ perinatal‐derived subgroup expressing invariant and semi‐invariant TCRs with no junctional diversity, which is made up by Vγ5^+^ DETC (in the skin) and the majority of Vδ6.1/Vδ6.3^+^ γδNKT cells (liver and spleen); and post‐natally generated cells expressing more polyclonal TCRγδ (mostly Vγ1^+^ or Vγ4^+^) with junctional diversity (localized in lymphoid tissues).
2. γδ17 T cells usually expressing Vγ6^+^ or Vγ4^+^ TCRs, although minor populations expressing Vγ1 and Vγ2/3 chains have been described in the thymus and most notably in the liver.^20^, ^21^ Vγ6^+^ γδ17 T cells are dominated by one invariant Vγ6Vδ1 clone lacking additional N‐nucleotide insertions and few semi‐invariant Vγ6Vδ1 clones,^7^, ^22^, ^23^, ^24^ whereas Vγ4^+^ γδ17 T cells present a more oligoclonal population encompassing multiple (semi)invariant TCRs.^22^, ^25^

In this review, we will focus on the biology of effector γδ T cells making IFNγ or IL‐17, from their early steps of differentiation in the thymus to their functional properties in the periphery, and the underlying molecular mechanisms.

## 2 QUEST FOR SURFACE MARKERS FOR MOUSE γδ T CELL EFFECTOR SUBSETS

Over the last decade or so, the field has put much effort into establishing markers to identify and analyze the two main effector γδ T cell subsets. The initial description of γδ17 T cells characterized them as CD3^high^CD4^−^CD8^−^CD45RB^low^CD44^high^CD62L^low^, implicating a memory‐like phenotype.^16^ It was therefore unexpected that peripheral γδ17 T cells did not express the IL‐2 and IL‐15 receptor common β chain (CD122) but the IL‐2Rα chain (CD25).^26^ By contrast, CD122 expression on γδ T cells correlated well with IFNγ production and thus became a marker for IFNγ‐producing γδ T cells (γδIFN T cells),^26^ later consolidated by Chien and colleagues.^27^ While CD122 and CD25 associated well with effector fates in the peritoneal cavity, it soon became clear that most γδ T cells in other tissues did not express either CD122 or CD25, although they produced considerable amounts of IFNγ or IL‐17 upon stimulation. By studying the tumor necrosis factor (TNF) receptor "superfamily" member CD27, our group described a robust marker differentially expressed on effector γδ T cell subsets in multiple tissues.^28^

CD27 had been previously used to characterize functional subsets of αβ T cells and natural killer (NK) cells.^29^, ^30^, ^31^ Among γδ T cells, in most tissues, CD27 was expressed by 70%‐90% of the cells, while 10%‐30% were CD27^−^ in the steady state. We found that CD27^−^ γδ T cells were CD44^high^CD62L^low^ and contained all IL‐17 producers but very few γδIFN T cells. In contrast, CD27^+^ γδ T encompassed most CD122^+^ γδ T cells, had lower expression of CD44 and produced IFNγ but essentially no IL‐17.^28^ Importantly, CD27 segregated two stable subsets in naïve mice, which retained their cytokine production characteristics upon in vitro culture and exposure to cytokines implicated in αβ T cell polarization, or upon adoptive transfer into lymphopenic recipients, even upon infection with malaria parasites (*Plasmodium berghei*). Most interestingly, we found that the effector phenotypes were already established during thymic development, and since embryonic life. Our detailed analysis of γδ T cell development revealed that manipulating CD70‐CD27 signals on developing γδ T cells in fetal thymic organ cultures (FTOC) impacted effector phenotype acquisition. In particular, CD27 signals were required for expression of the lymphotoxin‐β receptor (LTβR) and downstream genes previously associated with IFNγ production in γδ T cells.^32^ Consistent with this, CD27‐deficient mice showed a specific reduction in IFNγ (but not IL‐17) producers. Therefore, we established CD27 not only as a useful marker, but also a key regulator of γδ T cell functional differentiation.^28^ Similarly, Ly‐6C expression was described to subdivide effector γδ T cells, with γδIFN cells being Ly‐6C^+^ while γδ17 cells are exclusively Ly‐6C^−^.^33^ By comparing γδ T cells, based on CD44 and Ly‐6C expression, with CD8^+^ αβ T cells, the authors suggested γδ T cell naïve‐like and memory‐like subsets sharing characteristics with adaptive αβ T cells.^33^ Of note, the downregulation of CD27 has been documented when naïve γδ T cells encountered cognate antigen and underwent peripheral differentiation^34^ similarly to what has been reported for human antigen‐experienced/ memory γδ T cells.^35^, ^36^

**FIGURE 2:**
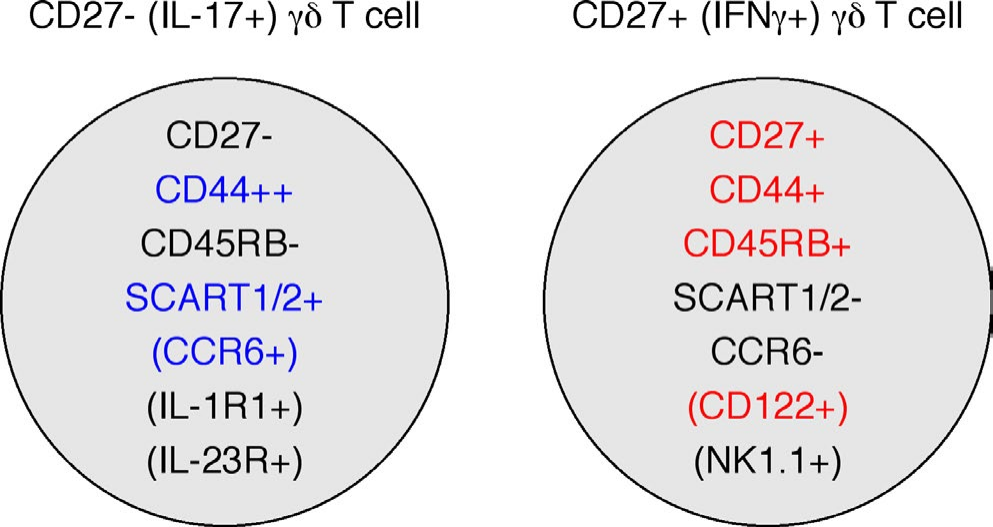
Expression status of cell surface receptors that segregate effector γδ T cell subsets. IL‐17^+^and IFNγ^+^γδ T cell subsets express different surface receptors enabling their identification and isolation based on these markers. The most commonly used markers are highlighted for IL‐17^+^γδ T (blue) and IFNγ^+^γδ T cells (red). Noted within brackets are surface markers expressed on particular subpopulations of the respective effector subsets

Besides lacking CD27 expression, γδ17 T cells were found to express SCART1 and SCART2 and CCR6 in the adult mouse.^37^, ^38^ CCR6 is the receptor for CCL20, a chemokine expressed upon microbial exposure, which has been reported to regulate lymphocyte migration towards sites of inflammation. The enrichment of γδ17 T cells among CCR6^+^ γδ T cells in the adult thymus and peripheral tissues was especially prominent and resulted in establishment of CCR6 as a γδ17 T cell marker. Of note, in the neonatal thymus only Vγ6^+^ γδ17 thymocytes express CCR6, whereas the vast majority of Vγ4^+^ γδ17 thymocytes do not; it was suggested that CCR6 expression might be induced extrathymically on dermal Vγ4^+^ γδ T cells.^39^ A detailed analysis of surface marker expression on γδ T cells in the neonatal thymus found that CCR6 was exclusively expressed on mature (CD24^−^) γδ T thymocytes.^20^ In the neonate, CD24^−^ Vγ4^+^ γδ T cells are mostly absent from the thymus. Therefore, it remains possible that CCR6 induction occurs intrathymically on Vγ4^+^ cells that promptly leave to peripheral sites. Interestingly, the expression of the two scavenger receptors SCART1 and SCART2 was shown to be mutual exclusive on Vγ6^+^ and Vγ4^+^ dermal γδ17 T cells, respectively, possibly due to different ontogenic origins.^40^ More recently, the combined use of CD24, CD44 and CD45RB allowed Pennington and colleagues to clearly identify the IFNγ (CD24^−^CD44highCD45RB^+^) and IL‐17 (CD24^−^CD44highCD45RB‐) committed subsets, as well as their developmental trajectories from immature (CD25^+^CD24^+^) γδ thymocytes.^20^ We find this phenotypic characterization of γδ T cells especially helpful (Figure 2).

Different thymic trajectories for the generation of γδIFN cells have been recently described by introducing transient surface markers, namely CD117, CD200 and CD371, as useful tools to segregate CD24^+^ γδ T cells within the adult thymus.^41^ CD371 identified the most immature γδ T cells; in the absence of TCR ligation during development, these cells matured to naïve (γδTn) cells with a gene expression profile similar to γδ17 cells, and were exported to the periphery still expressing CD24. In contrast, TCR ligation induced expression of CD200 and biased development towards γδIFN, with an intermediate stage marked by CD117 expression.^41^ This study emphasized the thymic export of CD24^+^ γδ T cells in adult mice; and confirmed that CD24^−^ γδ T cells in the adult thymus mostly represent long‐lived resident effector cells generated during fetal/ perinatal life.

## 3 THYMIC γδ T CELL DEVELOPMENT: PRE‐PROGRAMMING EFFECTOR FUNCTIONS

### 3.1 TCRγδ signaling – a driving force in effector cell commitment?

Among the surface markers that segregate the two γδ T cell effector subsets (described above; Figure 2), we encounter several proteins known as activation or maturation markers on conventional αβ T cells. In αβ T cells, activation markers are typically linked to TCR stimulation. Could the signal (strength) transmitted by the TCRγδ determine the acquisition of effector fate in γδ thymocytes?

A major difficulty in γδ T cell research has been the limited knowledge on specific TCRγδ ligands. Therefore, most of the early studies analyzing the impact of TCR signaling were carried out using TCR transgenic mice with known specificities, which did not allow to enquire the full γδ T cell repertoire and its TCR(Vγ)‐based subsetting described above. The synthesis of T10/T22‐tetramers (two closely related β2‐mircoglobulin‐associated nonclassical MHC class I molecules) for flow cytometry analysis enabled the first study of a minor but antigen‐specific γδ T cell subset within wildtype (ie non‐transgenic) mice.^42^ The analysis of T10/T22‐specific γδ T cells in wildtype and β2‐microglobulin‐deficient mice found that these γδ T cells developed in both mice and, consequently, antigen encounter was neither required nor inhibitory for their generation.^27^ Interestingly, the expression of maturation markers on T10/T22‐specific γδ T cells was affected in β2‐microglobulin‐deficient mice. While T10/T22‐specific γδ T cells in wildtype mice included cells expressing high levels of CD122 and were enriched for IFNγ producers, this subset was not detected in T10/T22‐specific γδ T cells of β2‐microglobulin‐deficient mice. Both mouse strains developed CD122^low^ T10/T22‐tetramer‐specific γδ T cells which produced predominantly IL‐17. Therefore, this study suggested that γδ17 T cell commitment was independent of ligand encounter during development, whereas the IFNγ‐producing fate required TCR engagement in the thymus.^27^

A subsequent study from our laboratory has provided additional data supporting a role of TCR signaling in the development of specific γδ T cell effector subsets. The research was based on a new mouse model, haploinsufficient for both CD3γ and CD3δ (termed CD3DH, for CD3 double haploinsufficiency), where we found both TCRγδ surface expression and TCRγδ signal strength to be substantially reduced.^43^ Consistent with this, thymic CD3DH γδ T cells had reduced expression levels of the activation/ maturation markers CD122, CD44, CD73 and CD5. A detailed analysis of thymic γδ T cells throughout ontogeny revealed that Vγ6^+^ γδ17 T cells were decreased in early life but Vγ4^+^ γδ17 T cells developed normally, resulting in normal γδ17 T cell abundance in the periphery of adult mice. In contrast, γδIFN T cells were diminished throughout life in the thymus and in peripheral lymphoid organs of CD3DH mice. Most importantly, CD122^+^ NK1.1^+^ γδIFN T cells (mostly Vγ1^+^) were virtually absent, but their thymic development could be rescued upon injection of an agonist CD3 antibody. The reduction in peripheral γδIFN T cells in CD3DH mice associated with less susceptibility to *P berghei* ANKA‐driven experimental cerebral malaria, an inflammatory syndrome dependent on IFNγ and γδ T cells.^43^, ^44^

The CD3DH phenotype is surprising considering that single haplodeficient mice did not show a similar impairment, and because several studies on TCR composition reported that CD3δ was not incorporated into the murine TCRγδ at least on mature γδ T cells.^45^, ^46^ Still, there might be a role of CD3δ subunit expression levels on TCRγδ assembly and expression during thymic development yet to be elucidated.

It is also intriguing that CD122^+^NK1.1^+^ γδIFN T cells appear to be especially dependent on TCRγδ signaling during development. It may be that these CD122^+^NK1.1^+^ γδ T cells represent a unique subset that has yet to be fully explored; we next summarize additional findings that support this possibility. In the thymus of adult wildtype mice, CD122^+^NK1.1^+^ γδ T cells are found among the CD44^high^CD24^low^ mature γδ T cells (also termed “cluster B”).^47^ Cluster B in adult mice is made up of two mature γδ T cell subsets, the γδIFN NKT cells and the γδ17 T cells, both thought to represent resident cells originating from an early developmental wave. Indeed, about 50% express NK1.1, CD122 and CD27 and are strongly biased for Vγ1Vδ6.3 TCR expression, which identifies them as γδNKT cells of early ontogenic origin.^38^, ^47^ It has been suggested that γδNKT cells undergo strong agonist selection during development, with endogenous ligand‐mediated activation of the Vγ1Vδ6.3 TCR inducing promyelocytic leukemia zinc finger (PLZF) expression and effector maturation of these cells.^48^, ^49^ In the periphery, Vγ1Vδ6.3 γδNKT cells are most abundant in the liver. In the recent characterization of a conditional *Bcl11b* knockout mouse, two hepatic γδ T cell subsets with different developmental requirements were described: a NK1.1^+^CD5^−^ subset generated early in newborn mice producing exclusively IFNγ rapidly upon infection; and a NK1.1^−^CD5^+^ subset that comprised both IFNγ and IL‐17 producers.^50^ Interestingly, *Bcl11b* deficiency resulted in a complete loss of the NK1.1^−^CD5^+^ γδ T cell subset (besides a general block in αβ T cell development), whereas the NK1.1^+^CD5^−^ proved to be Bcl11b‐independent and retained CD122, high PLZF expression and IFNγ production upon stimulation. These observations suggest that the NK1.1^+^ γδIFN T cell subset has specific developmental properties and represents a unique innate‐like population.

Another important clue on the role of TCR signaling in effector γδ T cell differentiation came from the analysis of Skint‐1‐deficient mice. Vγ5Vδ1 TCR‐expressing DETC were shown to rely on encountering Skint‐1 during development in the embryonic thymus to embark on the IFNγ effector program. Absence of Skint‐1 expression in a naturally occurring mutant strain resulted in Vγ5Vδ1 T cells that atypically produced IL‐17 instead of IFNγ.^51^ The experiments suggested that TCR ligation‐induced signaling was the driving force to switch away from an intrinsic fetal γδ17 T cell fate.

A cell‐intrinsic program indeed appears to drive the development of γδ17 T cells in the fetal/ perinatal thymus. Thus, IL‐17 expression in early precursors prior to TCR rearrangement has been documented in the fetal thymus; and γδ17 T cells appear to require only weak or even no TCR signaling for their development.^9^, ^52^ Moreover, strong TCRγδ stimulation in fetal thymic organ culture (FTOC) drastically reduced the generation of γδ17 T cells.^20^ It is worth to note that FTOC provide population‐based endpoint analyses but do not allow following single thymocytes during development. Therefore, it cannot be ruled out that specific cells/ populations are lost from the pool rather than undergoing fate switching.

As a final point on TCR signaling, several studies have reported variable expression and dependency on associated proteins by different γδ T cell subsets. The main Src family kinase Lck was surprisingly poorly expressed in mature γδ17 T cells in the thymus^40^ (and our unpublished observations), while Syk deficiency negatively impacted especially on γδ17 T cells^53^; and Blk was required specifically for Vγ6^+^ γδ17 T cell development.^54^ These observations may also indicate that there is more heterogeneity among developing γδ T cells (even at the signal transduction level) than initially expected.

### 3.2 γδ T cell progenitors – common or different for the effector subsets?

The identification of pre‐programmed γδ T cell effector subsets and their key contributions to peripheral immune responses highlighted, once more, the importance of understanding thymic development. How γδ T cell effector subsets acquire their effector functions during thymic development remains an area of active research and controversies, particularly whether γδ17 and γδIFN T cells arise from common or distinct thymic progenitors.^52^, ^55^ To understand the argument, we need to start at the αβ/γδ T cell bifurcation during early thymocyte development.

In the adult mouse, bipotential thymocytes were found up to the DN (double negative/CD4^−^CD8^−^) stage 2 (DN2), in which they simultaneously rearranged and transcribed *Trb*, *Trg* and *Trd*,^47^ whereas by the DN3 stage, the two lineages had fully diverged, as assessed through in vitro developmental potential assays.^56^, ^57^ Two types of model have been proposed for the bifurcation of the αβ and γδ T cell lineages: pre‐commitment and instructive models. A pre‐commitment model posits that a developing thymocyte is already set on its path/ fate when the TCR is expressed; and only cells that express a TCR concordant with its pre‐determined fate (ie, pre‐TCR for αβ T cells, and TCRγδ for γδ T cells) are able to develop further, while mismatched cells die off. On the other hand, an instructive model argues that the precursor is bipotential, and is directed into one or the other T cell lineage based on the TCR signals it receives, namely on the strength of such signals (reviewed in 58).

In support of pre‐commitment, cell heterogeneity within the DN2 subset has been described and linked to different lineage biases *prior* to TCR expression. One notable example is the expression of the interleukin 7 receptor (IL‐7R), since intrathymic injection and reconstitution of fetal thymic lobes with purified DN2 populations from adult mice demonstrated that the progeny of IL‐7R^high^ DN2 cells presented a higher γδ T cell to αβ T ratio compared to that of IL‐7R^low^ DN2 cells.^59^ Furthermore, IL‐7R signaling has been clearly implicated in the regulation of gene rearrangement and transcription at the *TCRγ locus*,^60^, ^61^, ^62^ while it may play additional roles in promoting the survival of γδ lineage cells.^63^, ^64^ Similarly, SRY‐related high mobility group box transcription factor 13 (Sox13) was identified as a putative γδ T cell‐specific lineage regulator in the thymus.^65^ Subsequent studies showed that Sox13 is required exclusively for thymic development of Vγ4^+^ γδ17 T cells, as these were absent in a spontaneous Sox13 mutant mouse strain, while other γδ T cell subsets appeared to develop normally.^66^, ^67^

On the other hand, TCR signal “strength” emerged as a key determinant based on studies using transgenic TCRγδ expression, in which the manipulation of downstream signaling mediators had major effects on γδ vs αβ T cell commitment. In such settings, γδ T cells required stronger TCR signals to develop than their αβ T cell counterparts.^68^, ^69^ It is noteworthy that besides full “knock‐out” (KO) mice, also conditional KO strategies have been used to assess the role of signaling proteins during development and activation of T cells. Conditional KO strategies rely on introduction of a *loxP* site flanked version of the gene of interested and the expression of *Cre* recombinase from a specific promoter whose activity is restricted to a certain cell type or tissue. The heterogeneity within the γδ T cell lineage and our incomplete understanding of its developmental trajectories impose limitations to the use of several conditional KO mice for the analysis of γδ T cells. Indeed, conditional deletion using the *proximal promoter of Lck*‐driven *Cre* (*pLckCre*) was shown to occur in DN2 cells and to efficiently target the αβ T cell lineage,^70^, ^71^ but had complex effects on γδ T cells: while it failed to delete floxed genes in most adult‐derived γδ T cells, it was significantly more efficient (in mediating deletion) in γδ T cells generated during early life, including DETC, γδ17 T cells and γδNKT cells.^72^ These data further suggest that the developmental pathways of distinct γδ T cell subsets may differ significantly.

An interesting possibility is the existence of various points of divergence from the αβ T cell lineage path for discrete γδ T cell subsets. Support for such a “multiple branching‐off model” first came from studies on E17 fetal thymi. While DN2 cells expressing high levels of c‐kit developed into both IFNγ^+^ and IL‐17^+^ γδ T cells, c‐kit^low^ DN3 cells failed to give rise to γδ17 T cells.^73^

More recently, a progenitor fate analysis and single‐cell RNA sequencing of discrete DN1 subpopulations, as previously defined by Petrie and colleagues,^74^ suggested that Vγ4^+^ γδ17 T cells derive from CD44^+^CD25^−^CD24^+^c‐Kit^−^ (DN1d) but not DN2 precursors.^52^ Such DN1d cells (from neonatal and young mice) contained a large proportion of Sox13‐expressing cells, previously linked to Vγ4^+^ γδ17 T cell development.^66^, ^75^ Spidale et al showed that DN1d cells had a transcriptional profile similar to that of Vγ4^+^ γδ17 T cells, which was inferred to be TCR‐independent from the analysis of mutant mice.^52^ In the original analysis of DN1d cells by Petrie and colleagues, it had been noted their poor proliferative potential and faster differentiation kinetics when compared to other DN1 and DN2 subsets. Moreover, the adult DN1d cell population contained no canonical T cell progenitors, showed B‐cell potential in vitro, and was therefore dismissed from being an intermediate between the common lymphoid progenitor and DN2 thymocytes.^74^

In contrast with the conclusions of Spidale et al,^52^ the earlier study by Shibata and colleagues reported that c‐Kit^high^ DN2 cells can develop into γδ17 T cells.^73^ Of note, the two teams analyzed thymocytes at different ages, which could explain the contradictory findings. Shibata et al used purified E17 thymocytes and analyzed total γδ17 T cells, which contain many Vγ6^+^ cells, whereas Spidale et al employed DN1 and DN2 precursors from 10‐day‐old thymi, which were introduced into FTOC, and focused on Vγ4^+^ γδ17 T cell development. Since γδ17 T cell thymocyte development is supposed to be terminated in 10‐day‐old mice,^9^ the question remains whether those DN1d progenitors are potential “leftovers” from the perinatal phase, which are “reset” for γδ17 T cell development upon transfer into the embryonic (E15) thymic environment of the FTOC. Thus, it would be informative to gain experimental evidence from the late fetal/ perinatal phase on Vγ4^+^ γδ17 T cell development excluding a DN2 stage, while directly assessing the differences to earlier fetal Vγ6^+^ γδ17 T cell development.

In sum, there may be two separate waves of γδ T cell development that differ substantially with regard to thymic precursors and dependence on TCR signaling. The first wave in the fetus is made up by a set of progenitor cells whose origin is not yet fully elucidated, but some reports suggest they may arise in the yolk sac.^10^, ^52^ These fetal progenitors are the first to develop in the thymus, an organ dynamically changing its composition and providing different microenvironments to developing T cells.^76^, ^77^ Indeed, the first γδ T cells, expressing TCR Vγ chains that follow the timing of chromatin opening along the *Tcrg* locus, may develop into IL‐17 producers by default, with no or low TCR signaling involved – unless the rearranged TCRs encounter their ligands, as it is the case for Vγ5Vδ1 DETC precursors, and potentially for Vγ1Vδ6 γδNKT cells. Ligand encounter does not seem to positively select the TCR repertoire, but instead divert the effector fate towards IFNγ production. Later on, a second wave of T cell progenitors enters the thymus; these differentiate towards γδIFN cells upon TCR signaling but are no longer prone to differentiate towards a default γδ17 fate, instead generating naïve, uncommitted γδ T cells that can display adaptive‐like behavior in the periphery.^41^, ^76^

Next, we will focus on the current view of the molecular mechanisms that underlie the acquisition and maintenance (or potential change) of γδ T cell effector functions.

## 4 γδ T CELL DIFFERENTIATION: FROM CHROMATIN TO POST‐TRANSCRIPTIONAL REGULATION

The acquisition of the capacity to secrete IFNγ and IL‐17 during thymic development distinguishes γδ T cells from their αβ T helper cell counterparts, whose effector cell differentiation (Th1 or Th17, in this case) occurs in peripheral lymphoid organs upon activation in specific inflammatory milieus.^78^ Thus, it became important to decipher the molecular mechanisms of γδ T cell differentiation, and assess how similar or different they are from those previously established for CD4^+^ T helper cells.

Some of the key questions addressed over the past decade have been:

1. How peripheral γδ T cell subsets expressing IFNγ or IL‐17 compare at the molecular level to their Th1 and Th17 counterparts, respectively;
2. Whether the molecular determinants identified in peripheral γδ T cell subsets are imprinted during thymic development;
3. What are the relative contributions of epigenetic, transcriptional and post‐transcriptional (including microRNA‐based) mechanisms to the regulation of IFNγ and IL‐17 expression in effector γδ T cell subsets.

In the next sections, we review the currently available data that provide some answers to these broad and still outstanding questions.

### 4.1 Epigenetic regulation of effector γδ T cell differentiation

Epigenetic mechanisms operating at the chromatin level control the maintenance of transcriptional networks ensuring autonomous maintenance of lineage phenotypes in differentiated cells, even through mitotic divisions.^79^ A very important of such epigenetic mechanisms is based on histone modifications, which can either associate with active gene expression or with gene repression. Thus, histone acetylation, H3K4 (lysine 4 of histone 3) methylation (H3K4me2 and H3K4me3) and H3K36me3 marks are associated with active transcription,^80^, ^81^ whereas H3K27me3 and H3K9me3 marks are deposited on silenced genes.^81^ Some of the first studies on genome‐wide distribution of histone marks were actually performed on T cells.^81^, ^82^ By correlating histone modifications with gene expression profiles, such studies constituted the basis of our current understanding of the epigenetic (histone‐based) landscape of active, repressed regions or “poised” genes. The latter, also called bivalent, show both active and repressive marks, mainly H3K4me2/me3 and H3K27me3, respectively.^83^ This poised state enables silenced genes to be rapidly induced under particular conditions, with gain of active histone marks and loss of repressive marks, therefore allowing developmental or functional plasticity. This is the case for CD4^+^ T helper cell subsets, where genes encoding transcription factors exhibit a large spectrum of epigenetic marks and allow for functional plasticity.^83^

Recurring to CD27 levels to segregate IFNγ^+^ and IL‐17^+^ γδ T cells, we isolated CD27^+^ (γδ27^+^) and CD27^−^ (γδ27^−^) γδ T cells, respectively, from peripheral organs of C57BL/6 mice and subjected them to Chip‐seq (chromatin immunoprecipitation followed by deep sequencing) analysis of activating H3K4me2 and repressive H3K27me3 marks.^84^ By comparing with Th1 and Th17 populations generated in vitro, we found that most of the genes differentially marked between γδ27^+^ vs γδ27^−^ T cell subsets were not segregating between Th1 and Th17 cells, suggesting that lineage‐specific mechanisms operate in γδ T cell differentiation.^83^, ^84^ An example was Dkk3, a modulator of Wnt signaling with known regulatory functions in CD8^+^T cells,^85^ which displayed active H3K4me2 marks selectively in γδ27^−^ T cells but not in γδ27^+^ T cells nor in either CD4^+^ helper T subset.^84^ Interestingly, Dkk3^−/−^γδ27^−^ T cells were enriched for IL‐17 producers when compared with wildtype controls, although the underlying mechanism is yet to be clarified.

**FIGURE 3:**
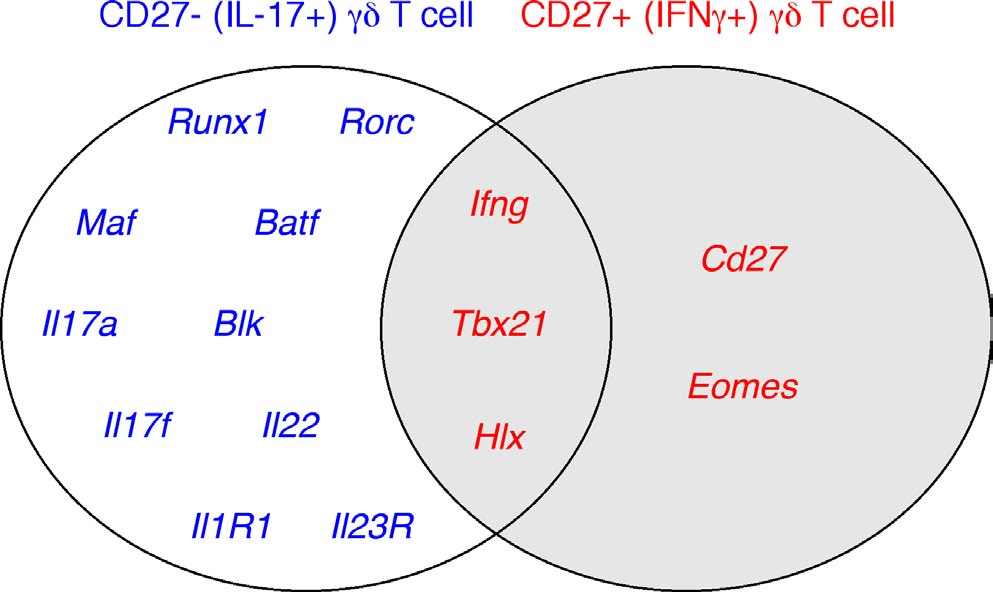
Epigenetically “active” gene loci associated with γδ T cell effector functions. CD27^−^and CD27^+^γδ T cells, corresponding to IL‐17^+^and IFNγ^+^γδ T cell subsets, respectively, display active dimethylated H3K4me2 marks in loci of genes associated with their effector functions (from the Chip‐Seq analysis in^84^). Loci of genes associated with IL‐17 production (blue) are only actively marked in CD27^−^T cells, whereas loci of genes related with IFNγ production (red) are actively marked in both subsets, highlighting the potential of IL‐17^+^γδ T cells to engage in both cytokine programs

Our detailed analysis of chromatin marks in genes involved in global γδ T cell biology revealed that those implicated in γδ T cell development (*Bcl11b*, *Id3 or Etv5*) and survival (*Actb*, *Bcl2*, *Bcl2l1*, *B2m*) displayed, as expected, very similar histone marking in γδ27^+^ and γδ27^−^ T cells.^84^ However, the pattern of active vs repressive marking in genes implicated in the cytokine production programs was unexpected. In particular, we were surprised to find that *Ifnγ* and its transcriptional regulators *Tbx21*, *Eomes* and *Hlx*, were all “primed” for expression in both γδ T cell subsets (Figure 3). By contrast, the presence of active histone modifications (H3K4me2 and H3 Acetylation) on *Il17a* and its key regulators, such as *Rorc*, *Blk* or *Batf*, as well as other type 17 signatures, like *Ccr6*, *Il1r1* and *Il23r*, was clearly restricted to γδ27^−^ T cells. Consistent with this chromatin landscape, the transcript levels of *Il17a* were substantially different (>1000‐fold) between the two subsets, whereas *Ifng* was only mildly (<10‐fold) higher in γδ27^+^ cells compared to γδ27^−^ cells. Importantly, the key epigenetic and transcriptional signatures observed in peripheral γδ T cell subsets were also present in their thymic counterparts,^84^ thus reinforcing the concept of developmental programming of effector γδ T cell subsets in the thymus.^27^, ^55^

Building on these molecular analyses, we challenged the functionalities of γδ T cell subsets in vitro and in vivo. We found that while γδ27^+^ cells do not produce IL‐17, even under strong type 17‐driving conditions, γδ27^−^ T cells can co‐express IFNγ (with IL‐17) in highly inflammatory settings, ie, upon stimulation with high amounts of IL‐1β and IL‐23, or in an ovarian cancer microenvironment.^84^ These data suggested that in contrast to the “hard‐wired” IFNγ producers within γδ27^+^ T cells, IL‐17‐producing γδ27^−^ cells are endowed with functional plasticity, which can be deployed (to co‐express IFNγ) under quite specific conditions. Subsequent work from our group showed that the polyfunctional γδ27^−^ (γδ17) T cell population can also secrete IL‐17F, IL‐22 and GM‐CSF upon IL‐1β and IL‐23 stimulation,^86^ which is fully consistent with the permissive landscape of this subset.^84^ Likewise, within CD4^+^ T cells, Th17 cells are substantially more prone to plasticity than their Th1 or Th2 counterparts. Thus, Th17 cells are able to transdifferentiate into Th1, regulatory T (Treg) and follicular helper T (Tfh) cells,^87^ with chromatin‐based mechanisms, including bivalent marks in master regulators like *Tbx21* or *Gata3*, also underlying their functional plasticity.^83^ Of note, Th17 cell plasticity is much more evident than that of γδ17 T cells. For example, under the IL‐23‐rich inflammatory environment of experimental autoimmune encephalomyelitis (EAE), Th17 converted into Th1 cells, whereas γδ17 T cells remained “pure” IL‐17 producers, ie they did not acquire IFNγ expression.^88^ This limited γδ T cell plasticity and its underlying mechanisms will be addressed at a later point (see 4.3.) in this review.

### 4.2 Transcriptional control of effector γδ T cell subsets

Following the genome‐wide analysis of γδ T cell subsets,^84^ our group assessed the specific roles played by “master” transcription factors, like T‐bet and RORγt, in controlling IFNγ and IL‐17 production in these lymphocyte populations.

T‐bet deficiency was found to significantly reduce IFNγ expression by peripheral γδ27^+^ T cells both in vitro and in vivo upon infection with murid herpes viruses, while also impairing IFNγ production by γδ27^−^CCR6^+^ T cells stimulated with IL‐1β or IL‐23 in vitro or during *Listeria* infection in vivo.^86^ By contrast, Eomes was not essential, since no differences in IFNγ expression were observed in Eomes^−/−^ γδ27^+^ cells compared to controls,^86^ which confirmed T‐bet as the main regulator of IFNγ expression by γδ T cells.^89^

On the other hand, RORγt (*Rorc*) deficiency completely abolished the production of IL‐17 by γδ27^−^ T cells.^86^ While in CD4^+^ T cells, IL‐17 production had been shown to be co‐regulated by the auxiliary transcription factors RORα and BATF,^90^ these factors were not required for IL‐17 production by γδ T cells, since the corresponding mouse KO models displayed normal γδ17 T cell numbers.^86^ The same was true for IRF4, shown to be dispensable for IL‐17 production by γδ T cells and other innate‐like lymphocyte lineages.^91^

Of relevance, in γδ T cells, an additional and critical layer of TF‐mediated regulation occurs during thymic development,^92^ and has been analysed in a global perspective by the Immgen consortium, that performed gene expression profiling of thymic γδ T cell subsets.^75^ This genome‐wide analysis identified three clusters of immature γδ T cells associated with distinct effector programs: the IL‐17 producers (Vγ6^+^ and Vγ4^+^), the IFN‐γ producers (Vγ1^+^, V7^+^) and DETCs (Vγ5^+^), with *Rorc, Maf*, *Sox13*, and *Sox4* associated with the IL‐17A producers; and *Tcf7* (TCF‐1), *Lef1, Tbx21* (T‐bet) *and Eomes* with the IFNγ producers. These results were confirmed in additional studies that showed, for example, that Sox13, Sox4, and the Ets family member ETV5, are key regulators of the development of γδ17 T cells.^66^, ^67^ Additionally, Hes‐1, a component of the Notch‐signaling pathway, and RelB, a member of NF‐κB family, promote IL‐17 production by γδ thymocytes,^93^, ^94^ while ID3 antagonizes this program by binding to HEB, an E protein TF required for expression of Sox13 and Sox4.^95^ Of note, although Eomes expression levels are consistently higher in γδIFN compared to γδ17 cells, this TF is not essential for γδIFN cell differentiation,^86^ as mentioned above.

A recent study has added an important temporal dimension to the role of some of these transcription factors regulating thymic γδ17 cell differentiation. Upon performing single‐cell analyses of *Sox13*, *Maf* and *Rorc* knockout mice, Sagar and colleagues have shown a sequential activation of these factors during both fetal and adult γδ17 cell differentiation.^96^ More specifically, Sox13‐deficient mice lacked Maf^+^ Rorc^+^ Il17a^+^ Il17f^+^ γδ T cells in the fetal thymus, and displayed reduced levels of Maf, Blk and Roc in γδ T cells from adult thymus, whereas Maf‐deficient fetal thymi lacked Rorc^+^ Il17a^+^ γδ T cells, and Rorc‐deleted γδ T cells did not show reduced Sox13 or Maf expression. Thus, during thymic γδ17 cell development, Sox13 acts upstream of c‐MAF which is essential for RORγt function in orchestrating the γδ17 program.^96^

A more detailed discussion on the transcriptional networks operating in effector γδ T cell subsets, especially during thymic pre‐programming, is provided by Anderson and colleagues elsewhere in this issue. Of note, an important regulatory function of TFs is to act as chromatin remodelers, mostly as promoters of open chromatin, thus leaving specific T cell loci accessible to other TFs/regulatory factors. Although, so far, no studies have specifically addressed this issue in γδ T cells, several TFs have been implicated in chromatin remodelling during CD4^+^ T helper cell differentiation. This is the case for BATF and IRF4, which promote opening of chromatin at Th17 cell‐specific loci, allowing access to RORγt.^90^ Also, members of the STAT family of TFs were shown to shape the landscape of Th1, Th2 and Th17 cells, with STAT1 and STAT4 inducing T‐bet, which then acts in conjunction with Eomes, Hlx, and Runx to induce Th1 cell differentiation, while STAT3 promotes Th17 cell differentiation.^83^, ^97^, ^98^ Although the role of STAT1 and STAT4 in the differentiation of IFNγ^+^ γδ T cells has not been addressed, STAT3 was shown to be dispensable for the generation of IL‐17‐producing γδ T cells.^93^ Therefore, the relevance of this layer of regulation (chromatin remodeling) for effector γδ T cell differentiation remains unclear.

Other issues that require further elucidation are (i) which extracellular cues, including potential TCR ligands, may feed in and regulate the transcriptional programs required for effector γδ T cell differentiation; and (ii) how the transcriptional networks described separately for thymic and peripheral γδ T cells are integrated, and potentially cross‐talk, within the cell. Future studies, based on single‐cell analysis, will likely contribute to improving our understanding of these phenomena.

### 4.3 Post‐transcriptional regulation of γδ T cell plasticity

Our group has been recently addressing the role of non‐coding RNAs, with special attention to microRNAs, in effector γδ T cell differentiation.

MicroRNAs (miRNAs) constitute a fundamental layer of post‐transcriptional regulation, acting as negative regulators of expression for most mammalian genes by promoting the degradation of mRNAs or preventing their translation.^99^ The overall relevance of miRNAs for immune cell functions has been demonstrated upon specific depletion of key enzymes involved in the biogenesis of miRNAs in early stages of immune cell differentiation. Namely, ablation of all mature miRNAs at early stages of thymocyte development via genetic deletion of Dicer or Drosha, two crucial enzymes in miRNA biogenesis,^100^ results in a developmental block that reduces numbers of peripheral mature αβ T and invariant natural killer T (iNKT) cells. Moreover, CD4^+^ T cells show reduced proliferation and survival after in vitro stimulation, but increased frequencies of IFNγ producers, implicating miRNAs in T helper cell differentiation.^101^, ^102^, ^103^ Furthermore, the absence of microRNAs in naïve T cells hinders the development and function of regulatory T cells (Tregs), thus disrupting the balance between effector and regulatory T cells, breaking tolerance and causing immune pathology in vivo.^101^, ^103^, ^104^

Interestingly, the development of γδ T cells is not impaired by miRNA ablation; on the contrary, there is a substantial increase of γδ T cells in the double negative thymic compartment of mice conditionally lacking Dicer in early thymocytes.^105^ However, only a very limited number of studies have addressed the role of miRNAs in γδ T cell differentiation. miR‐133b and miR‐206 were the first miRNAs shown to be co‐regulated with IL‐17 in both γδ and αβ T cells, but had no functional impact on cytokine production.^106^ In another study, based on the fact that miR‐181a/b‐1 cluster is highly expressed during thymocyte development and positively regulates TCR signal strength,^107^, ^108^ Prinz and co‐workers assessed its role in γδ T cells, but found miR‐181a/b‐1 deficiency to have no impact on thymic γδ T cell numbers or differentiation towards and IL‐17‐ or IFNγ‐producing effectors.^109^

**FIGURE 4:**
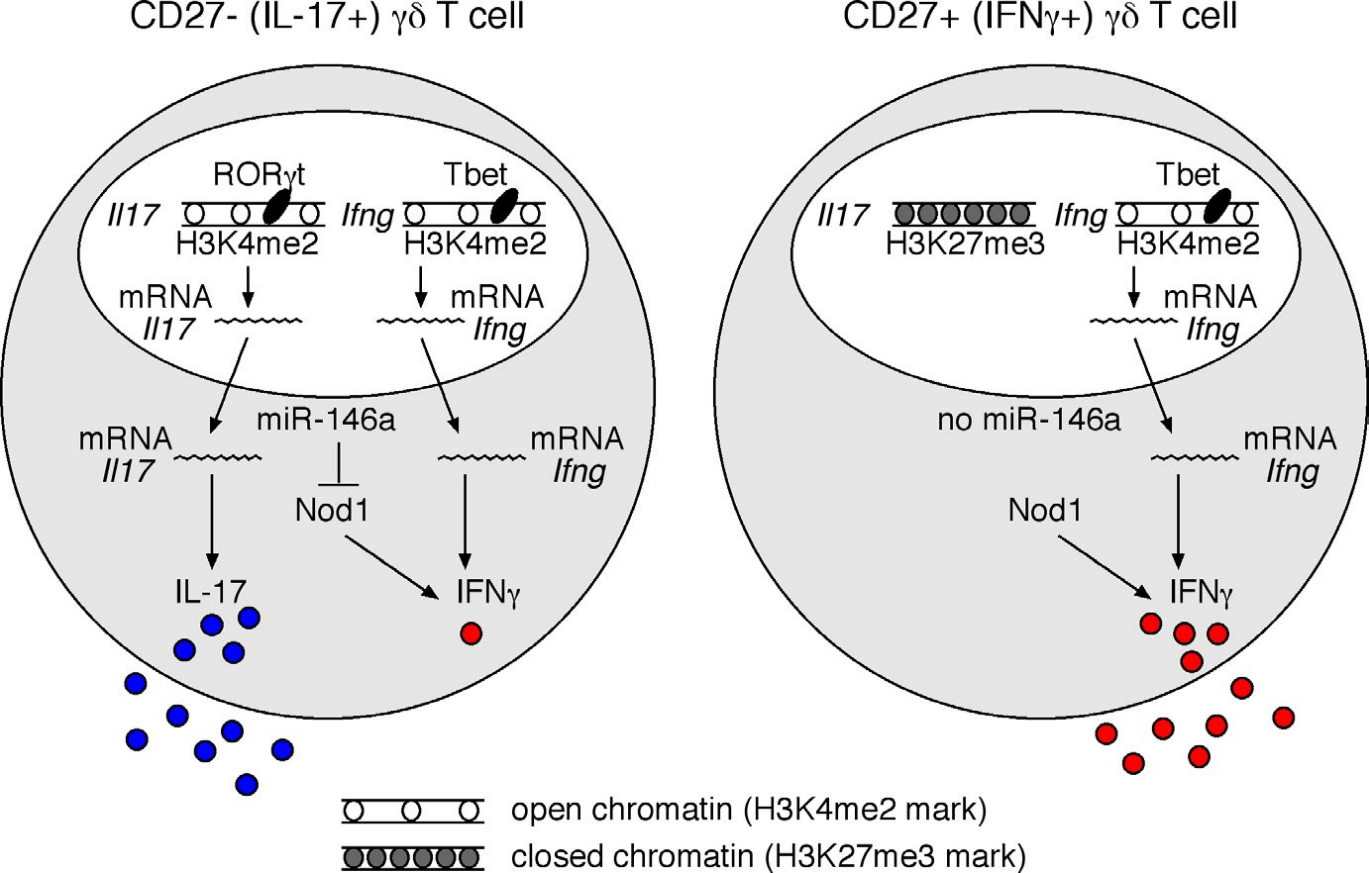
Regulation of IL‐17 and IFNγ expression in γδ T cell subsets. Integration of different levels of gene expression in CD27^−^(IL‐17^+^) and CD27^+^(IFNγ^+^) γδ T cell subsets. CD27^+^IFNγ‐producing γδ T cells display active marks (H3K4me2) exclusively in IFNγ and related transcription factor loci, while the IL‐17 locus is repressed (as dictated by repressive H3K27me3 marks). By contrast, CD27^−^IL‐17‐producing γδ T cells display active chromatin marks (H3K4me2) in both IL‐17 and IFNγ gene loci, as well as their respective transcriptional regulators RORγt and T‐bet, which drive IL‐17 and IFNγ mRNA expression, respectively. However, co‐production of IL‐17 and IFNγ is only observed under limited and strongly inflammatory conditions. In the steady state, CD27^−^IL‐17‐producing γδ T cells express high levels of miR‐146a, which acts as a brake for IFNγ production by inhibiting the expression of Nod1, an inducer of IFNγ production in γδ T cells

More recently, our group identified miR‐146a as functionally relevant for γδ T cell differentiation, and an important determinant of the limited functional plasticity of γδ27^−^ cells (Figure 4). Based on microarray analysis, we identified 35 miRNAs differentially expressed between γδ27^+^ and γδ27^−^ T cells from peripheral organs.^110^ MiR‐146a and miR‐146b were both upregulated in γδ27^−^ cells, but the later showed very low abundance in all T cell subsets analysed, and was therefore not studied further. MiR‐146a had previously been shown to exert anti‐inflammatory functions, including via regulation of the IFNγ program in both CD4^+^ and CD8^+^T cells.^111^ Consistent with the profile observed in peripheral γδ T cells, miR‐146a displayed markedly increased expression levels as thymic precursors matured into γδ27^−^ thymocytes.^110^ Interestingly, miR‐146a expression could not be modulated neither by exogenous TCR stimulation nor inflammatory cytokines, thus suggesting a tight control by endogenous thymic signals. Functional overexpression studies showed that miR‐146a down‐regulates IFNγ production in γδ27^+^ T cells. Conversely, in the miR‐146a KO mouse model,^111^ a cell‐intrinsic increased frequency of double‐producing IL‐17^+^ IFNγ^+^ cells was observed among γδ27^−^ T cells in vitro (stimulated with IL‐1β and IL‐23) and in vivo, upon *Listeria monocytogenes* infection.^110^

A differential Argonaute‐2 (Ago2) RIP‐seq strategy allowed us to identify Nod1 as a novel target of miR‐146a in γδ T cells,^110^ distinct from the conventional miR‐146a targets (Traf6, Irak1, and Stat1, among others) identified in other immune cells such macrophages, B cells and αβ T cells,^111^, ^112^, ^113^, ^114^, ^115^, ^116^ thus highlighting lineage‐specific roles for miRNAs based on the potential target transcripts that are present. We found Nod1 expression levels to be consistently lower in γδ27‐T cells (and Vγ1^−^Vγ4^−^/Vγ6^+^ γδ T cells) compared to γδ27^+^T cells, in accordance with the expected inverse correlation between the levels of a given miRNA and its target mRNA. In fact, the high expression of Nod1 in γδ27^+^ T cells is consistent with Nod1‐mediated promotion of IFNγ production in CD4^+^ and CD8^+^ T cells, given that Nod1‐deficient αβ T cells have impaired IFNγ responses in vivo.^117^ Furthermore, we found that Nod1^−/−^ γδ27^−^ cells were unable to differentiate into IL‐17^+^ IFNγ^+^ double producers, in opposition to the phenotype (accumulation of IL‐17^+^ IFNγ^+^ cells) of miR‐146a^−/−^ γδ27^−^ cells (all compared to WT controls).^110^ Moreover, by performing a genetic rescue experiment upon crossing miR‐146a^−/−^ with Nod1^−/−^ mice (analyzing littermate controls) revealed that heterozygous Nod1 reduction (in miR‐146^−/−^ Nod1^+/−^ mice) prevented the accumulation of double producers among γδ27^−^ T cells observed in miR‐146^−/−^ Nod1^+/+^ mice. These results collectively indicate that Nod1 is the key miR‐146a target implicated in the regulation of γδ27^−^ cell plasticity. Of note, until now, only one other miRNA has been implicated in T helper cell plasticity, miR‐10a, which restricts regulatory CD4^+^ T cells from acquiring Th17 and follicular helper T cell characteristics.^118^

In sum, we have shown that miR‐146a limits γδ T cell plasticity by targeting Nod1, an intracellular pattern recognition receptor that is an important mediator of endoplasmic reticulum (ER) stress‐induced production of pro‐inflammatory cytokines,^119^ although the specific mechanism of action in γδ T cells remains to be elucidated. Beyond miR‐146a, our group is committed to characterize other relevant miRNAs, and even the full miRNome of effector γδ T cell subsets.

### 4.4 Extracellular signals that control peripheral effector γδ T cell subsets

Peripheral effector γδ T cell responses rely either on the activation and expansion of thymically (fetally/ perinatally) pre‐programmed cells; or on activation and de novo differentiation of effectors from naïve γδ T cells exported from the adult thymus.^120^ While, barring a few exceptions,^34^ antigen‐specific γδ T cell responses remain illusive (as does the molecular identity of most TCRγδ ligands), various extracellular signals conveyed by co‐receptors and cytokine receptors have been implicated in controlling the peripheral pools of effector γδ T cell subsets.

Our group has shown that the CD70^−^CD27 pathway, besides critical during γδ thymocyte development,^28^ selectively promotes peripheral IFNγ^+^ γδ T cell responses via expansion of γδ27^+^ cells.^121^ CD27 signalling, which synergizes with TCR signals via non‐canonical NF‐κB, is required for survival and proliferation of γδ27^+^ cells, thus controlling their responses to viral and parasitic infections in vivo.^121^ CD28 is another co‐receptor that supports TCR signalling in promoting γδ T cell survival and proliferation, in this case via induction of IL‐2 production.^122^, ^123^ This is seemingly important for both γδIFN and γδ17 cells, since CD28‐deficient mice failed to expand both subsets upon *Plasmodium berghei* infection, which contrasted with the γδIFN‐specific effect of CD27.^121^, ^122^, ^123^

Other “costimulatory” (or inhibitory) receptors reported to differentially impact on γδIFN and γδ17 T cells are CD137, CD30, BTLA and PD‐1. Agonist anti‐CD137 (4‐1BB) antibodies promoted the expansion of IFNγ^+^ Vγ1^+^ T cells, which protected mice from *Listeria* infection in an IFNγ‐dependent manner.^124^ On the other hand, CD30‐deficient mice displayed a selective depletion of IL‐17^+^ Vγ6^+^ T cells in mucosal tissues in the steady‐state and upon *Listeria* infection, which associated with reduced bacterial clearance, and could be rescued upon administration of an agonist anti‐CD30 antibody.^125^ As for BTLA and PD‐1, they were shown to be important negative regulators of dermal γδ17 cells, with major pathophysiological impacted on psoriatic‐like skin inflammation.^126^, ^127^

Another important molecular layer of γδ T cell activation and differentiation are cytokines, including usual suspects like IL‐2^122^ or IL‐7,^128^ but especially the innate cytokines IL‐1β and IL‐23, which are pivotal for γδ 17 T cells. Indeed, stimulation with IL‐1β and IL‐23, but not TGF‐β or IL‐6, is sufficient to trigger abundant IL‐17 secretion by γδ27^−^ cells in vitro, even in the absence of TCR stimulation.^86^, ^129^, ^130^ This “innate” mode of γδ17 T cell activation is underlined by their rapid response to pathogen‐associated molecular patterns like lipopolysaccharide or lipoproteins, ligands for TLR4 and TLR2, respectively, via myeloid cells producing the key IL‐1β and IL‐23 cytokines.^121^ Notably, IL‐1β and IL‐23 are also the main inducers of γδ17 T cell plasticity, ie the co‐expression of IFNγ with IL‐17 in γδ27^−^ T cells,^84^, ^86^ as discussed above.

Finally, IL‐1β and especially IL‐23 can also drive de novo differentiation of γδ17 cells from naïve peripheral γδ T cells. While the acquisition of the IL‐17‐producing capacity of “natural” or thymic γδ17 T cells occurs exclusively during fetal/ perinatal development,^26^ relying on fetal progenitors^9^ and fetal thymic microenvironment,^131^ inflammatory IL‐1β and IL‐23 signals were found to induce the differentiation of peripheral γδ17 T cells from adult bone marrow‐derived naïve precursors.^132^, ^133^ This was shown not only in vitro but also in animal models or multiple sclerosis ^130^ and psoriasis.^133^ More recently, Zarin et al demonstrated that IL‐1β and IL‐23 also supported differentiation of γδ17 T cells in an in vitro OP9‐DL4 co‐culture system and in FTOC, suggesting an unanticipated role for these inflammatory cytokines during thymic development.^134^, ^135^

## 5 OPEN QUESTIONS AND PERSPECTIVES

This review focused on several layers of regulation of mouse effector γδ T cell differentiation, while highlighting our group's main contributions. Despite the significant progress made over the past decade, various issues remain incompletely understood.

The signals involved in dictating effector cell commitment continue to be an area of intensive research, now benefiting from full transcriptomic comparisons and pseudotime alignments of single thymocytes through the use of single‐cell RNA sequencing approaches. These may allow the dissection of novel TCR‐dependent vs independent thymic γδ T cell developmental pathways, as well as resolve the contradiction of having low or no TCR signalling in γδ17 T cell development within a γδ T cell lineage promoted (at the αβ/γδ bifurcation) by strong TCR signals. Moreover, such single‐cell approaches will permit further validation of the proposed model suggesting that γδ17 and γδIFN T cells arise from distinct thymic progenitors.^52^, ^55^

Besides the TCR, for which the identification of *bona fide* ligands remains a priority,^136^ it will be important to clarify which other molecular cues determine effector γδ T cell commitment, especially for γδ17 T cells for which TCR signals seem irrelevant (or even counterproductive.^20^ Recent evidence suggest that Notch signaling promotes γδ17 T cell development,^134^, ^135^ but it would be important to clarify the role of other major signaling axis, such as Wnt and Hedgehog.^137^ Ongoing studies to dissect the transcriptome and proteome of single thymocytes at different developmental stages will help to gain more insight into the trajectories and decision‐making during development of the heterogeneous γδ T cell lineage, taking into account ontogenic timing, distinct progenitor pools and the highly dynamic thymic microenvironment.

Following the seminal research performed on the basis of surface markers or TCR Vγ chain usage, we believe future studies should employ cytokine reporter mice to isolate pure populations of IL‐17‐ or IFNγ‐expressing cells, so that cellular and molecular properties can be directly associated to effector functions (within heterogeneous γδ T cell subsets). We have generated mice with reporter gene markers for both cytokines (*il17a‐*GFP *Ifng‐*YFP), which we are using to define the full mRNA and miRNA transcriptomes of “pure” effector γδ T cell subsets. By combining with single‐cell technologies it will be possible to enquire the potential heterogeneity even within IL‐17‐ or IFNγ‐expressing populations.

Another critical aspect will be how to best assess the function of particular genes within the γδ T cell lineage. Indeed, a major limitation of our (and other group's) studies so far is the analysis of γδ T cell phenotypes in full KO animals, rather than having specific gene ablation in γδ T cells. Although some promoters, such as *Lck* or *Cd4*, have been used to delete genes in the T cell compartment, in what refers to γδ T cells, the conditional KO strategy that ensures the most efficient deletion of genes is based on the use of *Vav1* promoter‐driven Cre (*Vav1*Cre).^72^ Thus, future studies should use this strategy to test gene requirements within the γδ T cell lineage. Ideally, one would use a highly penetrant lineage‐specific promoter, but that quest has turned out very difficult, with even *Tcrd‐*Cre showing limitations to delete genes in most γδ T cell populations (except DETC).^138^

Upcoming research should further elucidate the extracellular signals that drive peripheral effector γδ T cell responses. A current view that requires more experimental support is that γδ17 cells respond to innate signals, whereas γδIFN cells may be involved in adaptive‐like responses, including antigen specificity (for which, again, the identification of TCRγδ ligands remains critical). This is an outstanding topic also in human γδ T cell biology.^139^ On the other hand, with γδ17 cells being found to play important roles in steady‐state tissue physiology,^8^, ^140^, ^141^, ^142^, ^143^ the signals that regulate their activities in situ are now also a notable unresolved question.

Finally, while this review was focused on IL‐17 and IFNγ production as hallmark effector functions of murine γδ T cells, one should highlight their versatility in mice and humans: besides being highly cytotoxic, as acknowledged for decades and currently being explored for cancer immunotherapy,^144^, ^145^ γδ T cells can provide a pleiotropy of factors, from antimicrobial peptides ^146^ to wound healing associated cytokines like amphiregulin.^147^ Therefore, we believe the modulation of γδ T cell activities will hold promise in multiple settings of infection and inflammation‐associated diseases.

## CONFLICT OF INTEREST

The authors declared no conflicts of interest.
